# Perivitelline Space Abnormalities as Predictors of Fertilization and Embryo Quality in Intracytoplasmic Sperm Injection (ICSI) Cycles: A Prospective Observational Study

**DOI:** 10.7759/cureus.108288

**Published:** 2026-05-05

**Authors:** Vinodhini Rumesh, Vembu Radha

**Affiliations:** 1 Department of Reproductive Medicine and Surgery, Sri Ramachandra Institute of Higher Education and Research (SRIHER), Chennai, IND

**Keywords:** embryo quality, fertilization rate, intracytoplasmic sperm injection, oocyte morphology, perivitelline space

## Abstract

Background: Oocyte morphology is an important non-invasive indicator of developmental competence in assisted reproductive technology (ART). Among extra-cytoplasmic features, perivitelline space (PVS) abnormalities have shown inconsistent associations with fertilization and embryo quality after intracytoplasmic sperm injection (ICSI). This prospective observational study evaluated the association of PVS abnormalities with fertilization rates and embryo quality in ICSI cycles.

Methodology: Ninety-six ICSI cycles comprising 631 metaphase II (MII) oocytes were included. Oocytes were classified into four groups based on PVS morphology: normal PVS, large PVS (PVS-L), granular PVS (PVS-G), and combined PVS-L with PVS-G. Fertilization rates and day 3 embryo quality, graded according to the Istanbul Consensus criteria, were compared across groups using the chi-square test. Odds ratios (ORs) were calculated to assess the association with poor-quality embryos.

Results: Fertilization rates were highest in normal PVS oocytes (147/172, 85.4%) and lowest in PVS-L oocytes (132/177, 74.5%), although differences were not statistically significant (p = 0.082). Embryo quality was significantly associated with PVS morphology. Grade I embryos were most frequent in the normal PVS group (88/147, 59.9%) and least frequent in the combined PVS-L + PVS-G group (15/89, 17.1%) (p < 0.001). Oocytes with combined PVS abnormalities had a markedly higher risk of producing poor-quality embryos (74/89, 83.1%) compared with normal PVS oocytes (59/147, 40.1%).

Conclusion: PVS abnormalities, particularly when multiple abnormalities coexist, are associated with significantly poorer embryo quality in ICSI cycles. Assessment of PVS morphology may provide a valuable non-invasive adjunct for evaluating oocyte competence, although clinical outcomes such as pregnancy and live birth were not assessed in this study.

## Introduction

Intracytoplasmic sperm injection (ICSI) has become a cornerstone of assisted reproductive technology (ART), particularly for the management of severe male factor infertility and complex reproductive disorders. Despite advances in ovarian stimulation protocols, micromanipulation techniques, and embryo culture systems, fertilization failure and impaired embryo development remain clinically significant challenges in ICSI cycles [1]. Among the various factors influencing ART outcomes, oocyte quality plays a central role in determining fertilization success, embryo developmental competence, and implantation potential [2,3]. Recent evidence has further refined the understanding of oocyte morphometry and its relationship with developmental competence in ART [4]. Consequently, non-invasive morphological assessment of oocytes at the time of denudation has gained increasing attention as a practical and cost-effective method for optimizing embryo selection. However, the strength of evidence supporting specific morphological features remains debated [5, 6].

Morphological evaluation of mature metaphase II (MII) oocytes includes assessment of both intra-cytoplasmic and extra-cytoplasmic characteristics, such as cytoplasmic granularity, polar body morphology, zona pellucida integrity, and the perivitelline space (PVS) [7]. While several studies have investigated the prognostic significance of cytoplasmic dysmorphisms, the clinical relevance of extra-cytoplasmic abnormalities, particularly those involving the PVS, remains less clearly defined [8, 9]. The PVS, located between the oolemma and the zona pellucida, plays an important role in physiological processes including polar body extrusion, cortical granule exocytosis, and sperm-oocyte interaction, all of which are essential for normal fertilization and early embryogenesis [10].

Among the various PVS abnormalities identified during routine embryological assessment, an abnormally large PVS (PVS-L) and the presence of granular or particulate debris within the PVS (PVS-G) are most frequently observed [11]. These morphological alterations may result from premature cortical granule release, altered zona pellucida dynamics, cytoplasmic shrinkage, or degenerative changes within the ooplasm [12]. Such alterations are hypothesized to reflect underlying cytoplasmic dysmaturity or cellular stress, which may adversely affect spindle formation, pronuclear development, and subsequent embryo cleavage [13].

Previous studies examining the association between PVS morphology and ICSI outcomes have produced inconsistent findings [1, 14, 15] due to differences in PVS classification criteria, observer subjectivity, and variability in ovarian stimulation protocols and laboratory conditions. A recent systematic review specifically focusing on perivitelline space debris concluded that while PVS abnormalities are associated with poorer embryo quality, their independent predictive value remains uncertain [16].

Importantly, most available studies have focused on isolated oocyte-level observations or mixed cohorts in which morphologically normal and abnormal oocytes coexist within the same cycle. This limits the ability to determine the independent prognostic value of PVS abnormalities [7, 9]. Furthermore, relatively few studies have systematically evaluated the relationship between PVS morphology and embryo quality using standardized grading systems, despite embryo morphology being a critical surrogate marker for implantation potential [17].

Given the ongoing debate and limited prospective data, well-designed studies are needed to evaluate the impact of PVS abnormalities on fertilization and embryo quality in ICSI cycles. A clearer understanding of this relationship may enhance the clinical utility of routine oocyte morphological assessment as a non-invasive biomarker of oocyte competence and support more informed embryological decision-making. Therefore, this prospective observational study aimed to evaluate the association between perivitelline space abnormalities (normal PVS, PVS-L, PVS-G, and combined PVS-L+PVS-G) and fertilization rates and embryo quality in ICSI cycles. The hypothesis was that oocytes with PVS abnormalities, particularly combined abnormalities, would show lower fertilization rates and poorer embryo quality compared with oocytes having normal PVS morphology.

## Materials and methods

Study design and setting

This prospective observational study was conducted in the Department of Reproductive Medicine and Surgery at Sri Ramachandra Institute of Higher Education and Research (SRIHER), Chennai, India, over one year from May 2024 to May 2025. Consecutive couples undergoing ICSI as part of ART were enrolled in the study.

Study population

Cycles involving controlled ovarian hyperstimulation followed by ICSI were eligible for inclusion. Exclusion criteria included cycles using surgically retrieved sperm, preimplantation genetic testing, severe oligoasthenoteratozoospermia, and advanced maternal age in order to minimize potential confounding related to severely compromised gametes. Advanced maternal age (≥40 years) was excluded to minimize confounding associated with age-related oocyte aneuploidy and cytoplasmic deterioration, which could independently influence PVS morphology and embryo outcomes. Only cycles yielding MII oocytes suitable for morphological assessment were included in the final analysis.

Ovarian stimulation and oocyte retrieval

Controlled ovarian hyperstimulation was performed using recombinant follicle-stimulating hormone (rFSH) alone or in combination with human menopausal gonadotropin (HMG). The dosage was individualized based on female age, body mass index (BMI), ovarian reserve parameters, and previous ovarian response. A gonadotropin-releasing hormone (GnRH) antagonist protocol was used, with antagonist initiation when the leading follicle reached ≥14 mm. Final oocyte maturation was triggered using a GnRH agonist, human chorionic gonadotropin (hCG), or a dual-trigger regimen according to the institutional protocol. Transvaginal ultrasound-guided oocyte retrieval was performed 34 to 36 hours after trigger administration.

Semen analysis and sperm preparation

Semen samples were obtained by masturbation after two to 7 days of abstinence and analyzed according to World Health Organization (WHO) 2010 criteria [18]. Sperm preparation was performed using discontinuous density gradient centrifugation followed by swim-up or simple washing, depending on sperm concentration and motility. Morphologically normal and progressively motile spermatozoa were selected for injection.

ICSI procedure and fertilization assessment

Intracytoplasmic sperm injection was performed using standard micromanipulation techniques. Fertilization was assessed 17 ± 1 hours after injection. Normal fertilization was defined by the presence of two pronuclei (2PN).

Oocyte morphological assessment and PVS classification

Following enzymatic and mechanical denudation, all MII oocytes were evaluated under an inverted microscope for morphological characteristics. PVS morphology was assessed. Oocytes were classified into four groups: normal PVS, PVS-L, PVS-G, and combined PVS-L with PVS-G, based on previously described morphological criteria [1, 7]. Classification was based on PVS width and the presence of particulate debris. Oocytes were assigned to groups before outcome assessment. All oocyte morphology assessments were performed by a single experienced embryologist to maintain consistency. Interobserver reliability was not formally assessed, which is acknowledged as a limitation.

Embryo culture and grading

Fertilized oocytes were cultured under standard laboratory conditions. Embryo quality was assessed on day 3 after ICSI using the Istanbul Consensus grading system, which evaluates blastomere number, fragmentation percentage, cell symmetry, and multinucleation [17]. Embryos were categorized as Grade I (good), Grade II (fair), or Grade III (poor) for analysis.

Outcome measures

The primary outcomes were fertilization rate and embryo quality across different PVS morphology groups. Fertilization rate was calculated as the proportion of normally fertilized oocytes among the total number of injected oocytes. A secondary analysis evaluated the association between PVS morphology and poor embryo quality, defined as Grade II and Grade III embryos. For secondary analysis, Grade II and Grade III embryos were combined as ‘poor-quality embryos’ because both are associated with lower implantation potential compared with Grade I embryos, consistent with prior literature.

Statistical analysis

Data were analyzed using IBM SPSS Statistics software, version 20.0 (IBM Corp., Armonk, NY, USA). Categorical variables were expressed as frequencies and percentages. Group comparisons of fertilization rates and embryo quality were performed using the chi-square test. Odds ratios (ORs) with 95% confidence intervals (CIs) were calculated to assess the association between PVS abnormalities and poor embryo quality. A p-value less than 0.05 was considered statistically significant. Analyses were performed at the oocyte level without adjusting for clustering of multiple oocytes within patients or for potential confounders (e.g., maternal age, ovarian reserve, and infertility etiology). Only unadjusted estimates are presented. Multivariable analysis was not performed due to the exploratory nature of the study and the risk of overfitting. 

## Results

A total of 96 consecutive ICSI cycles comprising 631 metaphase II oocytes were included in the final analysis. The mean female age was 32.4 ± 5.0 years, and the mean BMI was 27.4 ± 5.3 kg/m². Regarding BMI categories, 30 (31.3%) women had normal BMI, 39 (40.6%) were overweight, and 26 (27.1%) were obese. Most couples had primary infertility (59, 61.5%), while 37 (38.5%) had secondary infertility. The mean duration of infertility was 5.8 ± 3.9 years. In terms of causes of infertility, female-factor infertility was observed in 38 (39.6%) couples, male-factor infertility in 26 (27.1%), combined factors in 22 (22.9%), and unexplained infertility in 10 (10.4%) (Table 1).

**Table 1:** Baseline characteristics of the study population (n = 96 couples)

Parameter	Value
Female age (years), mean ± SD	32.4 ± 5.0
BMI (kg/m²), mean ± SD	27.4 ± 5.3
BMI category, kg/m^2^, n (%)	
Normal (18.5–24.9)	30 (31.3)
Overweight (25–29.9)	39 (40.6)
Obese (≥30)	26 (27.1)
Type of infertility, n (%)	
Primary	59 (61.5)
Secondary	37 (38.5)
Duration of infertility (years), mean ± SD	5.8 ± 3.9
Cause of infertility, n (%)	
Female factor	38 (39.6)
Male factor	26 (27.1)
Combined	22 (22.9)
Unexplained	10 (10.4)

A total of 631 oocytes were evaluated for PVS morphology. Normal PVS was observed in 172 (27.2%) oocytes. Among abnormal PVS patterns, PVS-L was present in 177 (28.0%), PVS-G in 173 (27.4%), and the combined large and granular PVS (PVS-L + PVS-G) in 109 (17.2%) oocytes. Overall, abnormal PVS morphology accounted for the majority of oocytes, compared with those with normal PVS (Table 2).

**Table 2:** Distribution of oocytes according to perivitelline space (PVS) morphology (n = 631 oocytes)

Perivitelline space (PVS) morphology	Number (%)
Normal PVS	172 (27.2)
Large PVS (PVS-L)	177 (28.0)
Granular PVS (PVS-G)	173 (27.4)
PVS-L + PVS-G	109 (17.2)

Fertilization rates varied according to PVS morphology. The highest fertilization rate was observed in normal PVS (147/172, 85.4%), followed by PVS-L + PVS-G (89/109, 81.6%) and PVS-G (139/173, 80.3%), while the lowest rate was observed in PVS-L (132/177, 74.5%). However, the difference in fertilization rates among the groups was not statistically significant (p = 0.082) (Table 3).

**Table 3:** Fertilization rates according to perivitelline space (PVS) morphology

Perivitelline space (PVS) morphology	Fertilized/Total	Fertilization rate (%)
Normal PVS	147/172	85.4
Granular PVS (PVS-G)	139/173	80.3
Large PVS (PVS-L)	132/177	74.5
PVS-L + PVS-G	89/109	81.6
p-value: 0.082		

Grade I embryos were most frequent in the normal PVS group (88; 59.9%), followed by the PVS-G group (44; 31.6%), the PVS-L group (37; 27.9%), and the combined PVS-L + PVS-G group (15; 17.1%). The difference between groups was statistically significant (p < 0.001). Grade II embryos were observed in 44 (29.9%) oocytes with normal PVS, 59 (42.4%) with PVS-G, 51 (38.7%) with PVS-L, and 37 (41.6%) with combined PVS-L + PVS-G. However, the difference among groups was not statistically significant (p = 0.125). Grade III embryos were least frequent in the normal PVS group (15; 10.2%) and were more common in the PVS-G group (36; 26.0%), the PVS-L group (44; 33.4%), and the combined PVS-L + PVS-G group (37; 41.6%). This difference was statistically significant (p < 0.001) (Table 4).

**Table 4:** Embryo quality distribution by perivitelline space (PVS) morphology (n = 507 embryos)

Embryo grade	Normal perivitelline space (PVS) (n=147)	Granular PVS (PVS-G, n=139)	Large PVS (PVS-L, n=132)	PVS-L + PVS-G (n=89)	p-value
Grade I	88 (59.9%)	44 (31.6%)	37 (27.9%)	15 (17.1%)	<0.001
Grade II	44 (29.9%)	59 (42.4%)	51 (38.7%)	37 (41.6%)	0.125
Grade III	15 (10.2%)	36 (26.0%)	44 (33.4%)	37 (41.6%)	<0.001

Poor-quality embryos were observed in 59 (40.1%) cases in the normal PVS group. Higher proportions were noted in the PVS-G group (95; 68.4%), the PVS-L group (95; 72.0%), and the combined PVS-L + PVS-G group (74; 83.1%). Compared with the normal PVS group, the odds of poor embryo quality were significantly higher in the PVS-G group (OR: 3.23, 95% CI: 1.98 to 5.28), the PVS-L group (OR: 3.83, 95% CI: 2.32 to 6.31), and the combined PVS-L + PVS-G group (OR: 6.74, 95% CI: 3.56 to 12.8) (p < 0.001) (Table 5).

**Table 5:** Association between perivitelline space (PVS) morphology and poor embryo quality

Perivitelline space (PVS) morphology	Poor quality embryos n (%)	Odds ratio (95% CI)	p-value
Normal PVS	59 / 147 (40.1)	Reference	—
Granular PVS (PVS-G)	95 / 139 (68.4)	3.23 (1.98–5.28)	<0.001
Large PVS (PVS-L)	95 / 132 (72.0)	3.83 (2.32–6.31)	<0.001
PVS-L + PVS-G	74 / 89 (83.1)	6.74 (3.56–12.8)	<0.001

## Discussion

The present prospective observational study evaluated the impact of PVS abnormalities on fertilization and embryo quality in ICSI cycles. It demonstrated that deviations in PVS morphology are associated with inferior embryological outcomes. Although fertilization rates declined with increasing PVS abnormalities, the most pronounced and statistically significant effect was observed in embryo quality. A clear shift toward lower embryo grades was observed in oocytes with PVS-L, PVS-G, or both.

In the present cohort, oocytes with normal PVS morphology had the highest fertilization rate (85.4%), whereas those with PVS-L morphology had the lowest (74.5%). Although this difference did not reach statistical significance, a non-significant trend toward lower fertilization rates was observed in oocytes with PVS abnormalities. No definitive effect can be concluded from these data. Similar observations were reported by Ten et al. [14], who demonstrated significantly reduced fertilization rates in oocytes with widened PVS or PVS debris and attributed this effect to compromised cytoplasmic competence and impaired pronuclear formation. In contrast, earlier studies by De Sutter et al. [15] did not find a significant relationship between PVS morphology and fertilization outcomes, suggesting that mild PVS abnormalities may be physiologically tolerated during fertilization. These discrepancies across studies may reflect differences in classification criteria, study populations, and ovarian stimulation protocols, highlighting the need for standardized assessment methods.

Notably, the impact of PVS abnormalities became more evident when embryo quality was evaluated. In this study, Grade I embryos were significantly more frequent in the normal PVS group (59.9%) compared with the PVS-G (31.6%), PVS-L (27.9%), and combined PVS-L and PVS-G groups (17.1%). Conversely, Grade III embryos were markedly higher among oocytes with combined PVS abnormalities. This progressive decline in embryo quality across PVS categories suggests a potential dose-response relationship between the severity of PVS abnormality and impaired embryonic development. These findings are consistent with previous reports indicating that PVS irregularities reflect underlying cytoplasmic dysmaturity that may persist beyond fertilization and negatively influence early cleavage dynamics [1, 7, 13].

The biological plausibility of these findings is supported by studies implicating PVS abnormalities in disrupted cytoskeletal organization, altered mitochondrial distribution, and premature cortical granule exocytosis [12, 19]. A widened PVS may result from cytoplasmic shrinkage or abnormal zona pellucida elasticity, whereas granular debris within the PVS is believed to represent degenerative cytoplasmic fragments or apoptotic bodies [10, 12]. Such alterations may compromise spindle integrity and chromosomal alignment, leading to impaired embryo cleavage and increased fragmentation, as previously described by Alikani et al. [13] and Sciorio et al. [20].

The OR analysis further strengthens the clinical relevance of PVS assessment. Oocytes with combined PVS-L and PVS-G abnormalities demonstrated more than a six-fold increased risk of producing poor-quality embryos compared with oocytes with normal PVS morphology. This finding supports the concept that cumulative morphological abnormalities may have a synergistic negative effect on oocyte developmental competence. Similar conclusions were reported by Balaban and Urman [9], who emphasized that although isolated morphological abnormalities may be tolerated, the presence of multiple dysmorphisms significantly compromises embryo quality and implantation potential.

Importantly, no significant association was observed between PVS morphology and maternal age or BMI in the present study. This suggests that PVS abnormalities may reflect intrinsic oocyte-level factors rather than systemic demographic influences. This observation is consistent with previous studies reporting inconsistent correlations between patient characteristics and oocyte morphological features, further supporting the value of direct oocyte assessment during ICSI [7, 11].

From a clinical perspective, these findings highlight the potential value of incorporating PVS morphology into routine oocyte evaluation protocols. Although PVS abnormalities alone should not preclude the use of affected oocytes, their presence, particularly when combined, may provide additional information to support embryo selection, especially in cycles with limited embryo availability. However, caution is required when extrapolating these findings to implantation or pregnancy outcomes because the present study did not evaluate clinical pregnancy or live birth rates.

The strengths of this study include its prospective design, the use of standardized embryo grading based on the Istanbul Consensus criteria, and the comprehensive evaluation of both fertilization and embryo quality outcomes. Nevertheless, several limitations should be acknowledged. Most importantly, the absence of implantation, clinical pregnancy, and live birth data means that the clinical relevance of PVS abnormalities remains unknown. Embryo quality is only a surrogate marker, and conclusions about clinical utility cannot be drawn without these outcomes. Other limitations include the single-center design, which may limit generalizability, and the lack of formal interobserver reliability assessment. Additionally, PVS assessment remains subjective despite standardized criteria. Additionally, the analysis did not account for clustering of oocytes within patients or adjust for potential confounders using mixed-effects or multivariable models, which may affect the robustness of the findings. Further, the presented estimates are unadjusted. A multivariable model adjusting for confounders such as age, BMI, and infertility etiology would strengthen the findings. Future studies should employ such models to better isolate the independent effect of PVS morphology.

## Conclusions

PVS abnormalities in MII oocytes are associated with impaired embryological outcomes in ICSI cycles. Although fertilization rates showed a declining trend with increasing PVS abnormalities, embryo quality was significantly compromised, particularly when PVS-L and PVS-G abnormalities were present simultaneously. Assessment of PVS morphology may provide additional descriptive information about oocyte competence, but its clinical utility for embryo selection requires validation in studies that include pregnancy and live birth outcomes.
